# Synthetic and Tissue-Engineered Vascular Grafts: Current Status, Emerging Technologies, and Clinical Prospects

**DOI:** 10.31083/RCM45083

**Published:** 2025-12-23

**Authors:** Kejian Gong, Shixiong Wei, Xinyun Zhang, Wei Liu

**Affiliations:** ^1^Department of Thoracic Surgery, The First Hospital of Jilin University, 130021 Changchun, Jilin, China

**Keywords:** artificial blood vessels, material selection, fabrication methods, tissue-engineered vascular grafts

## Abstract

Cardiovascular diseases (CVDs) are the leading cause of morbidity and mortality worldwide, creating an urgent demand for small-diameter vascular substitutes with durable long-term patency. Large-caliber synthetic grafts, such as polyethylene terephthalate (PET) and ePTFE, are well established in clinical practice; however, these synthetic grafts fail in small-diameter applications due to thrombosis and intimal hyperplasia. Moreover, autologous grafts are constrained by limited availability and variable quality. Recently, synthetic degradable polymers (e.g., polycaprolactone (PCL), poly(lactic-co-glycolic acid) (PLGA)), and extracellular matrix-derived natural materials (collagen, gelatin, silk fibroin, bacterial cellulose) have drawn increasing attention, as each offers distinct advantages and limitations in terms of mechanics, biocompatibility, and degradation behavior. Meanwhile, emerging fabrication technologies, including electrospinning, thermally induced phase separation, microfluidic spinning, and three-dimensional printing, are advancing the structural biomimicry and functional optimization of artificial vascular grafts. Thus, building on these developments, this review further examines the design strategies of tissue-engineered vascular grafts (TEVGs), focusing on cell sourcing, *in vitro* and *in situ* endothelialization, antithrombotic modification, and the prevention of intimal hyperplasia, while also summarizing outcomes from preclinical models and early clinical trials. Despite promising progress, the widespread clinical translation of TEVGs remains limited by prolonged manufacturing cycles, high costs, and insufficient long-term patency. Hence, future efforts toward standardized cell sources, integrated structure, function design, and multicenter clinical validation are critical to the development of next-generation vascular grafts.

## 1. Introduction

Cardiovascular diseases (CVDs) are one of the most serious global public health challenges and are now the leading cause of death worldwide [1]. According to statistics from the World Health Organization, CVDs are responsible for approximately 17.9 million deaths each year [2]. They are associated with a variety of potential risk factors, including unhealthy lifestyle behaviors such as smoking and physical inactivity, as well as systemic conditions such as hypertension, hypercholesterolemia, hyperlipidemia, and diabetes [3]. In the context of atherosclerotic coronary artery disease, aortic pathologies, and other cardiovascular conditions, the native vasculature may become incapable of ensuring adequate tissue perfusion. Under such circumstances, surgical interventions—including vascular repair, replacement, or bypass grafting—are often required to restore blood flow. Globally, more than one million vascular bypass procedures are performed each year, and in the United States alone, the annual number of coronary artery bypass grafting (CABG) surgeries approaches 600,000, underscoring the substantial demand for vascular substitutes [4]. Synthetic materials are now widely applied in vascular grafting, with large-diameter (>6 mm) prosthetic grafts being used extensively in clinical practice. Such grafts typically achieve 5-year patency rates of 70–90% when implanted in aortic or iliac positions [5]. However, small-diameter prosthetic vascular grafts still face several challenges, such as early graft thrombosis and late-stage neointimal hyperplasia leading to luminal stenosis, with 2-year patency rates often falling below 30% [6]. Autologous vessels, such as the saphenous vein, radial artery and internal mammary artery, are therefore regarded as the first-choice of graft material for small-diameter vascular reconstruction due to their superior long-term patency and minimal risk of immunogenic rejection [7]. However, autologous vascular grafting also presents several limitations, including the restricted availability of donor vessels, suboptimal quality or pathology of the native vessels, and subpar long-term graft patency following transplantation [8, 9]. Although arterial bypass grafts demonstrate superior long-term patency and clinical outcomes, their use is limited by restricted anatomical length and technical difficulty in procurement [10, 11]. In contrast, venous bypass grafts, such as those using the great saphenous vein, often exhibit biomechanical incompatibility with the arterial circulation, leading to accelerated graft atherosclerosis. Clinical data indicate that saphenous vein grafts exhibit failure rates of 8–25% within the first postoperative year, and approximately 50% occlude or lose function within 10 years [12, 13, 14]. Therefore, continued research into the fabrication technologies and broad clinical applications of artificial blood vessels (ABVs), and in particular small-diameter grafts, is of great significance for advancing the field of cardiovascular surgery. While numerous studies have reported the outstanding properties of these technologies, there is a lack of comprehensive reviews that systematically summarize the developmental directions and clinical prospects in the field. This review will discuss material selection, fabrication strategies, and clinical limitations of polymer-based ABVs and tissue-engineered vascular grafts (TEVGs), with a particular emphasis on the precise development of small-diameter vascular grafts. Furthermore, it also discusses recent progress towards addressing these challenges. By directly dealing with the key issues and integrating recent advances, this review provides a deeper understanding of the developmental trajectory of the field.

## 2. Material Selection for Artificial Vascular Grafts

**Fig. 1.**
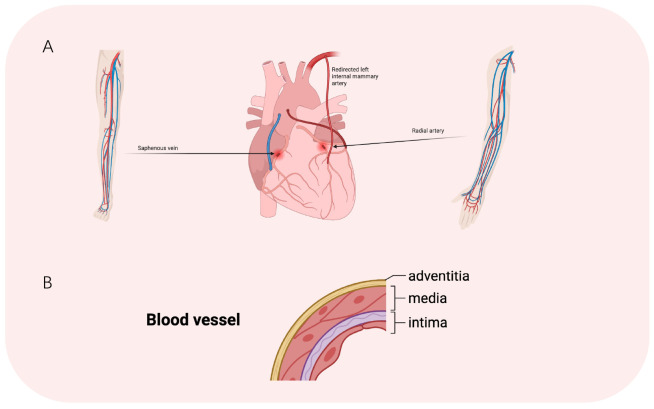
**Schematic illustration of CABG and structural composition of a blood vessel**. (A) Main options for autologous vascular substitutes. (B) Three-layer structure of blood vessels.

The circulatory system consists of a complex network of vessels of varying types and diameters, including the aorta and vena cava (25–30 mm), arteries and veins (0.6–16 mm), arterioles and venules (20–25 µm), and capillaries (approximately 9 µm) [15]. Anatomically, blood vessels are composed of three distinct layers: the intima formed by endothelial cells (ECs), the media composed of smooth muscle cells (SMCs), and the adventitia containing fibroblasts (Fig. 1) [16, 17]. The modern use of artificial vascular grafts is widely thought to have originated in 1952, when Arthur Voorhees first employed Dacron to replace the aorta in a canine model [18]. This was followed by the first successful human implantation in 1954 [18, 19]. To this day, polyester-based materials such as Dacron remain key components in the fabrication of synthetic blood vessels [20]. However, their inherent lack of biointegration, compliance, and regenerative capacity restricts their clinical application primarily to large-diameter vessels. These materials are still unable to fulfill the requirements for small-diameter grafts, particularly in terms of long-term functionality and patency [21, 22]. This section provides a comprehensive overview of the advantages and disadvantages of commonly used vascular graft materials, alongside recent advancements.

### 2.1 Materials for Synthetic Artificial Blood Vessels 

To overcome limitations of autologous vascular grafts, increasing attention is being paid to the development of synthetic polymer materials. Polymeric materials offer excellent tunability and outstanding mechanical properties, providing sufficient strength and rigidity in vascular engineering to withstand physiological blood pressure and shear stress. Among the materials in clinical use, polyethylene terephthalate (PET) and polytetrafluoroethylene (PTFE) have achieved satisfactory outcomes in large-diameter (>6 mm) vascular reconstruction [23].

PET and PTFE possess strong mechanical strength, excellent durability, tunable chemical structures, and superior processability, making them among the preferred materials for constructing ABVs [24, 25]. However, their hydrophobic surface limits the ability to interact with cells, leading to insufficient EC coverage. This increases the risk of thrombosis and reduces biocompatibility, thereby restricting their application in vascular grafts [16]. Current strategies to address the hydrophobic surface include modification of the surface and blending with other materials [26, 27]. Some surface modification techniques are widely used to endow ABVs with antithrombotic properties. Strategies such as grafting bioactive short peptide segments [28], nitric oxide (NO) donors [29, 30], and various drugs [31] can effectively exert anticoagulant effects and inhibit platelet adhesion. Multiple Antigen Peptide–Arginine-Glycine-Aspartic acid (MAP-RGD) modification achieved approximately 67% patency at 4 weeks in a rat model, showing a significant advantage over unmodified polycaprolactone (PCL)/collagen grafts (0%), with partial but incomplete endothelial regeneration observed in the lumen. Heparin-modified grafts demonstrated a patency of 64% at 1 month, but the endothelial coverage at 2 weeks was only 40%, comparable to that of untreated controls. In contrast, vascular endothelial growth factor (VEGF)-modified grafts exhibited 88% patency at 1 month and 82% endothelial coverage at 2 weeks, markedly outperforming the other groups. Overall, VEGF modification demonstrated more sustained patency and superior enhancement of endothelialization, highlighting its potential as a more promising surface modification strategy. Another approach to surface modification is to introduce hydrophilic functional groups such as –OH, –COOH, and –NH₂ through plasma treatment or surface functionalization, thereby enhancing cell adhesion and promoting spontaneous EC coverage of vascular grafts [32]. Blending of PET and PTFE with other materials is a more commonly adopted strategy by researchers. Some bioactive molecules with antithrombotic effects can also be loaded onto ABVs to significantly enhance their performance. Bivalirudin (BVLD) can directly block the active site of thrombin, thereby inhibiting downstream coagulation reactions and suppressing platelet adhesion and activation [33]. Xing *et al*. [34] improved the hydrophilicity of the material surface through the introduction of a polydopamine coating and used a peptide derived from the extracellular matrix (ECM), REDV (Arg-Glu-Asp-Val), in combination with BVLD to modify ePTFE vascular grafts. In a porcine model, the modified grafts were able to maintain patency and achieve endothelialization at 12 weeks post-implantation [34]. Traditional oil-based lubricants such as naphtha and isopar are commonly used during the fabrication of ABVs. However, these cannot dissolve water-soluble biomolecules like arginyl-glycyl-aspartic acid (RGD), heparin, and selenocystamine (SeCA). This limitation hinders their deep and uniform incorporation into the material and limits their ability to become biofunctional. To address this, researchers have recently explored the use of alcohol–water mixtures as alternative lubricants, resulting in significantly improved biological performance and safety of the vascular grafts [35].

Despite the widespread use of synthetic materials in fabricating vascular grafts, their limited biodegradability within the body often results in extended persistence that can subsequently lead to intimal hyperplasia, immune rejection, and infection. PCL and poly(lactic-co-glycolic acid) (PLGA) are two biodegradable materials that are widely used in scaffold systems [36, 37, 38], absorbable sutures [39], and drug delivery systems [40, 41, 42, 43]. PLGA is a copolymer of lactic acid (PLA) and glycolic acid (GA). It is characterized by a faster rate of degradation, which helps to reduce adverse reactions caused by foreign body stimulation [44]. Higher GA content in PLGA (e.g., 50:50) accelerates degradation but lowers local pH and may cause inflammation, whereas higher PLA content (e.g., 75:25, 85:15) slows degradation, prolongs support, and mitigates acid accumulation [45]. However, PCL degrades more slowly, thereby offering prolonged mechanical support and enhanced lumen stability and patency [46]. Nevertheless, these materials still face several challenges in practical applications. For example, the byproducts generated during degradation may be potentially toxic to surrounding tissues. In addition, the degradation rate of biodegradable vascular grafts may not meet the necessary requirements for mechanical support [16, 25].

### 2.2 Natural Materials for Artificial Blood Vessels 

Natural materials such as collagen, gelatin, chitosan, silk fibroin, and bacterial cellulose are also widely used in the construction of artificial blood vessels. These natural macromolecules are primarily components of the ECM and are derived from plant, animal, or human tissues. They exhibit excellent biocompatibility and cytocompatibility, effectively promoting cell adhesion and proliferation, thereby accelerating tissue repair and regeneration [47, 48]. As mentioned above, blood vessels are composed of three distinct layers, with the ECM playing a critical role in maintaining normal vascular function.

Collagen and elastin are major components of the ECM and provide structural strength and stiffness to native vessels. They are also amongst the most commonly used natural materials for fabricating ABVs [49]. Collagen exhibits excellent biocompatibility and is commonly used to construct vascular scaffolds, or to serve as the outer layer of ABVs [50]. It not only provides essential structural support, but also promotes cell adhesion and proliferation [51]. The inherent biodegradability of collagen allows it to be gradually absorbed and metabolized in the body, thereby reducing adverse reactions associated with long-term implantation. Collagen scaffolds can form hierarchical nanostructures and microarchitectures similar to natural ECM, thereby serving as temporary ECM to guide tissue regeneration [52, 53].

Gelatin (Gel) is a partially hydrolyzed derivative of collagen that retains many bioactive sequences, such as RGD, which enhance cell adhesion and proliferation. It exhibits good biocompatibility and is low-cost and easy to process, making it widely used in artificial vascular grafts [54]. However, its poor thermal stability and rapid degradability necessitate crosslinking or composite strategies to improve its mechanical strength and stability. For example, PET can form a stable bond with gelatin through an intermediate polydopamine coating, which remains stable under physiological conditions for up to 24 days and promotes the adhesion and spreading of ECs and SMCs [24].

Chitosan (CS) is a naturally occurring cationic polysaccharide enriched with amino and hydroxyl groups. Its outstanding biocompatibility, degradability, and antimicrobial activity have led to its extensive use in biomedical applications such as tissue repair and drug delivery [55]. Due to its cationic nature, CS can interact effectively with cells to enhance cell adhesion and promote the regeneration process. In addition, CS can form electrostatic complexes with anionic polysaccharides, synergistically improving the mechanical properties and biocompatibility of the material [56]. Based on these characteristics, Rodrigues *et al*. [57] constructed a stable, 3D scaffold microenvironment using CS that formed a physical hydrogel network through electrostatic interactions with alginate (ALG). This provided an ideal platform for the loading and release of functional proteins such as elastin-like recombinamers (ELRs), while the cationic surface of CS also promoted attachment of ECs [57].

Silk fibroin (SF) is a natural protein derived from silkworm cocoons [58]. In addition to good biocompatibility and biodegradability, SF microspheres are also capable of controlled drug release [59, 60]. Bacterial nanocellulose (BNC) is a cellulose-based biomaterial synthesized by bacteria and characterized by high crystallinity and low solubility. Compared to easily degradable biomaterials, BNC can provide prolonged structural support *in vivo*, thereby preventing vascular collapse caused by rapid degradation [61]. Bao *et al*. [58] embedded heparin-loaded SF nanoparticles onto the surface of BNC vascular grafts. This composite material exhibited superior anticoagulant properties and demonstrated enhanced endothelialization and anti-hyperplasia characteristics in subcutaneous implantation experiments in animals [58].

Although strategies involving natural materials in tissue engineering have continued to evolve, the development of biomimetic vascular grafts that simultaneously exhibit high strength, high elasticity, and excellent antithrombotic properties remains a significant challenge [62, 63]. Despite their favorable biocompatibility and bioactivity, natural materials often possess low structural density and lack hierarchical organization, resulting in insufficient mechanical properties to withstand the complex shear forces present in the *in vivo* hemodynamic environment [64]. Therefore, future research should investigate more advanced fabrication techniques to enhance the architectural hierarchy of engineered blood vessels and to improve their functional performance and long-term stability in clinical applications.

Overall, synthetic materials such as PET and PTFE possess excellent mechanical strength and durability, but their hydrophobic and bioinert surfaces result in insufficient endothelialization, requiring surface functionalization to reduce the risk of thrombosis. Biodegradable polymers such as PCL and PLGA provide tunable degradation properties and better compliance, with PCL offering prolonged support due to its slow degradation, while PLGA degrades more rapidly to facilitate tissue remodeling, though its acidic by-products may induce inflammation. In contrast, natural materials such as collagen and gelatin contain RGD motifs that strongly promote cell adhesion and proliferation, but their rapid degradation and poor mechanical stability restrict their use to luminal coatings or composite layers rather than load-bearing scaffolds. Chitosan, owing to its cationic nature, enhances cell adhesion and exhibits antibacterial properties, but also requires crosslinking or blending with other materials to improve strength. Silk fibroin demonstrates good cytocompatibility and moderate mechanical performance and can be used for controlled drug release, while bacterial nanocellulose provides excellent structural stability but lacks intrinsic bioactivity, thus requiring functionalization to improve hemocompatibility. In the future, material selection for small-diameter vascular grafts will need to rely on composite designs that balance mechanical stability with biological activity. Table 1 summarizes the quantitative mechanical data of natural–synthetic composite scaffolds and native human vessels, highlighting the differences in tensile strength and Young’s modulus between these materials.

**Table 1.** **Quantitative mechanical data of natural-synthetic composite scaffolds versus native human vessels**.

Materials	Tensile strength (MPa)	Young’s modulus (MPa)
PET	36	-
PTFE	2.91	5.75
PCL	1.49	13.69
Collagen	-	3.7–11.5
PCL/Gelatin	3.91	8.9
HA/Gelatin	0.97	0.56
PCL/SF	2.1	23.28
BNC	1.06	3.25
Coronary artery	1.5	-

PET, polyethylene terephthalate; PTFE, polytetrafluoroethylene; PCL, polycaprolactone; HA, hyaluronic acid; SF, silk fibroin; BNC, bacterial nanocellulose.

## 3. Fabrication Techniques for Artificial Blood Vessels

In recent years, an increasing number of advanced technologies have been applied to the construction of ABVs. In addition to traditional casting and spinning methods, emerging techniques such as electrospinning, 3D printing, and thermally induced phase separation (TIPS), as well as their combinations, have opened up new possibilities and research directions for the design and performance optimization of vascular grafts. This section summarizes the advantages and limitations of such technologies, and presents recent advances in their application to ABV research.

### 3.1 3D Printing

3D printing is an emerging technology capable of fabricating complex structures [65]. It allows the personalized construction of ABVs prior to surgery based on patient-specific imaging data [66, 67]. The primary materials used are bioinks composed of biocompatible polymers. These must meet rigorous criteria, including excellent biocompatibility, faithful replication of the native vascular ECM, and the ability to support cell adhesion, proliferation, and tissue regeneration [16, 68]. Biodegradable polymeric materials, including PLA, PLGA, and PCL, were a preferred choice for bioink formulation in previous studies due to their low cost and good biocompatibility [69]. However, as discussed in Section 2 of this paper, limitations such as insufficient mechanical strength, uncontrolled degradation rates, local effects of the degradation byproducts, and inherent hydrophobicity have restricted their application in ABVs.

ECM bioinks are a widely studied and innovative 3D printing strategy that aims to replicate the natural ECM by incorporating proteins, polysaccharides, and other biomolecules to support cell adhesion, growth, and tissue formation [70]. However, due to the inherent complexity and variability of ECM compositions from different tissue sources, the structural stability and longevity of ECM-based printed tissues require further evaluation through *in vivo* studies [71]. With the advance of bioprinting technology and the expansion of printable materials, researchers have explored the use of decellularized ECM (DECM) and cells in addition to polymers as possible bioinks [72]. DECM is derived from natural biological materials through physical or enzymatic decellularization processes, thereby preserving the native structure and function of the ECM [73, 74]. DECM effectively mimics the mechanical properties of native tissues, offering robust structural support and tensile strength that regulate cell behavior and promote functional tissue formation [75]. ABVs constructed using DECM can be customized for individual-specific applications, enabling more precise and targeted tissue replacement [76]. Jang *et al*. [77] successfully implemented DECM-based 3D bioprinting in 2017 by introducing mesenchymal stem cells (MSCs) and ECs as seed cells. This also provided significant support for the development of TEVGs. Cells used in 3D bioprinting are derived from diverse sources and can be applied either individually or in combination to better replicate the complexity of native tissue structures [78]. For cardiovascular tissue engineering, the cells must exhibit several essential characteristics, including high proliferative capacity, rapid maturation, strong differentiation potential, ease of acquisition, and low immunogenicity to the host [78, 79]. Stem cell engineering has shown great promise in the treatment of CVDs [80]. Human embryonic stem cells (hESCs) and human induced pluripotent stem cells (hiPSCs) can be induced to differentiate into various functional cell types, such as vascular ECs and SMCs [81, 82]. Notably, hiPSCs possess the capacity to generate the three-layered structure of blood vessels—including ECs, SMCs, and fibroblasts—making them particularly suitable for vascular tissue construction [78].

A variety of advanced 3D printing technologies have been widely adopted in the biomedical field, including inkjet-based bioprinting, laser-assisted bioprinting (LAB), extrusion-based bioprinting, acoustic bioprinting, coaxial bioprinting, and stereolithography (SLA)-based bioprinting [83]. Coaxial bioprinting utilizes a concentric dual-nozzle system to simultaneously extrude two different bioink formulations in a core–shell configuration, resulting in tubular structures with circumferentially layered architecture [84]. The outer shell serves to protect the inner core, while the layered extrusion approach allows the spatial separation of bioinks containing different cell types, facilitating generation of the three-layered structure of blood vessels [85, 86, 87]. Bosch-Rué *et al*. [87] developed bilayered hollow fibers by co-extruding two cell-laden hydrogels and a sacrificial polymer through a triple coaxial nozzle. Human umbilical vein ECs (HUVECs) were encapsulated in the inner layer, and human aortic SMCs (HASMCs) in the outer layer. Both cell types showed over 90% viability after extrusion and 20 days of culture, with alignment patterns mimicking native vessel structure, i.e., HUVECs parallel and HASMCs perpendicular to the vessel axis [87]. Qu *et al*. [88] reported a Filament Diameter-Adjustable 3D Printing (FDA-3DP) strategy. Compared to traditional 3D printing with direct ink writing (DIW), the FDA-3DP method enables dynamic adjustment of printing speed and nozzle height, allowing the fabrication of structures with controllable gradient porosity and without the need for equipment replacement [88].

Artificial intelligence (AI) has recently attracted significant attention in the biomedical field [89, 90]. For instance, the integration of AI can enhance the accuracy of tissue construction during bioprinting and assist in building complex *in vitro* models, as well as monitoring and analyzing cell growth [91]. However, the application of AI in tissue engineering is still in its early stages. The development of robust AI models is needed to effectively address the current limitations of 3D printing technologies, such as operational complexity and high costs [16, 92, 93].

### 3.2 Decellularization

Although DECM was introduced in Section 3.1 as a bioink for 3D printing, decellularization itself also represents an independent fabrication strategy for vascular grafts. Unlike its role as a material source, this approach directly employs whole native vessels as scaffolds by removing cellular components while preserving the natural ECM composition, three-layered structure, and vascular microarchitecture. The resulting decellularized scaffolds retain the mechanical properties and biochemical cues of native vessels, thereby providing an ideal platform for host cell repopulation and vascular remodeling [94]. However, the decellularization process may compromise biomechanical integrity and accelerate elastin deformation and degradation [95]. To address these limitations, decellularized scaffolds are often combined with other materials to restore mechanical strength. Gong *et al*. [96] reinforced decellularized aortic grafts by electrospinning PCL onto their outer surfaces. Scanning electron microscopy revealed severe damage to the medial layer of the decellularized vessel, while mechanical testing demonstrated that electrospun polycaprolactone (ES-PCL) significantly enhanced biomechanical performance. Moreover, vascular ultrasound and micro-CT angiography confirmed that the implanted grafts maintained satisfactory patency for up to six weeks in a rat model [96].

### 3.3 Casting and Spinning

Casting and spinning are among the earliest and most widely used techniques for fabricating ABVs. However, these traditional methods struggle to precisely control pore size and distribution [16]. Consequently, many researchers have focused on trying to improve these conventional processes. Electrospinning is a specialized spinning technique that uses an electric field and a collector electrode to eject a polymer solution from a tiny nozzle, forming a fibrous network with high porosity that resembles the ECM of human tissues, thereby promoting cell adhesion and proliferation [25, 97, 98]. Bioactive substances and drugs can be incorporated into ABVs via electrospinning to achieve improved biocompatibility [99], including gelatin [100], rapamycin [101], heparin [102] and VEGF [103]. Electrospinning is often combined with other techniques to achieve enhanced performance. Kuang *et al*. [104] combined electrospinning and freeze-drying to construct a poly(L-lactide-co-ε-caprolactone) (PLCL)-based vascular scaffold with sustained heparin release, which enhanced endothelial adhesion and inhibited SMC proliferation. In addition, improvements in electrospinning processing techniques can enable the fabrication of blood vessels with complex geometries to meet various challenging transplantation needs [105]. Moreover, vascular grafts with intricate structures can also provide anti-torsion properties and mimic the mechanical characteristics of native vessels. Native blood vessels possess unique mechanical properties due to the wavy arrangement of elastin and collagen fibers within their walls [106]. Yan *et al*. [107] fabricated a bilayer small-diameter vascular graft composed of an inner layer of eggshell membrane and an outer layer of thermoplastic polyurethane (TPU) using electrospinning with a wavy cross-sectional rotating collector. This distinctive wavy structure resulted in a nonlinear stress–strain response and demonstrated excellent graft repeatability under cyclic loading [107]. During the electrospinning process, organic solvents such as dichloromethane, tetrahydrofuran, and N,N-dimethylformamide are commonly used to facilitate material dissolution and fiber formation [108]. However, residual amounts of these solvents may remain during fiber fabrication due to incomplete evaporation, raising potential toxicity concerns when electrospinning is used to produce ABVs. Current research efforts to address this issue mainly focus on developing solvent-free or low-toxicity alternatives, such as melt electrospinning and aqueous electrospinning, and improving the processing parameters to enhance the efficiency of solvent evaporation [108, 109, 110]. Compared with solvent-based electrospinning, which typically produces fibers in the nano to submicron range (approximately 100–1000 nm), solvent-free electrospinning (melt/MEW) generally yields fibers in the micron scale (1–20+ µm). However, these fibers exhibit higher crystallinity and lower porosity, thereby enabling slower, more sustained drug release without an obvious burst effect. In addition, the absence of residual solvents makes them more suitable for long-term implantation applications. Additionally, techniques like freeze-drying and vacuum drying are employed to further reduce residual solvents. Nevertheless, more comprehensive toxicological and animal studies are needed to evaluate the safety and clinical applicability of electrospun ABVs [108].

Microfluidic spinning is also an increasingly popular spinning technique [111]. By precisely manipulating small volumes of fluid within microscale channels, this technique enables controlled fluid processing in minimal volumes and the reproduction of complex biological structures. It therefore has great potential in tissue engineering and organoid cultivation [112, 113]. Based on these features, Jia *et al*. [114] developed a microfluidic spinning approach using a coaxial glass capillary system. By adjusting the flow rate and composition of fluids in the microchannel, these authors successfully fabricated helical microfibers. The unique spiral structure induces swirling blood flow, which increases shear stress and effectively inhibits thrombosis formation. Microfluidic spinning technology enables researchers to modify vascular structures with greater precision, beyond simple straight or curved forms. This facilitates the creation of more controllable architectures that can be tailored to suit different types of blood vessels.

### 3.4 Thermally-Induced Phase Separation

The technique of TIPS induces phase separation between the solvent and polymer during cooling of a polymer solution, resulting in the formation of a porous structure. This method relies on temperature variation to promote polymer chain aggregation while expelling the solvent, thereby forming a 3D porous network. When combined with techniques such as electrospinning and braiding, TIPS can optimize both mechanical properties and biological functionality [115]. Ma *et al*. [116] reported a small-diameter vascular graft with a biomimetic three-layered structure in which a loose and porous PEFUU scaffold was constructed using TIPS. This porous architecture facilitated the formation of neovascular networks that exhibited superior mechanical performance compared to the control groups [116].

Overall, 3D printing technology can be tailored to fabricate complex vascular structures according to patient-specific needs, but its printing precision and the mechanical strength of bioinks still require improvement. Electrospinning enables the preparation of highly biomimetic fibrous networks that provide an ideal environment for cell adhesion; however, their small pore size hinders deep cellular infiltration, and the potential risk of residual toxic organic solvents remains a concern. Traditional casting and spinning methods are cost-effective and well established, but they lack precise control over pore distribution and structural complexity. Microfluidic spinning can generate specialized fibers that effectively prevent thrombosis, yet its mechanical performance and scalability remain limited. TIPS can rapidly produce porous scaffolds that facilitate tissue ingrowth, but its ability to finely control structural details is constrained. At present, the closest alternatives to native blood vessels are decellularized scaffolds. They preserve the natural three-layered structure and bioactivity, offering favorable compliance and biological guidance. Nevertheless, the decellularization process often compromises mechanical properties and leads to elastin degradation, while clinical applications continue to face challenges such as insufficient long-term patency and potential immune rejection.

## 4. Tissue-Engineered Vascular Grafts

TEVGs have attracted significant attention as an alternative for vascular transplantation. They are constructed using biodegradable polymers to form tubular scaffolds, which are then seeded with autologous cells and matured in a dynamically simulated physiological environment before being implanted into the body. Over time, the biodegradable components are gradually replaced by newly formed ECM, thus closely mimicking the properties of native blood vessels [117].

Weinberg and Bell [118] first proposed the concept of TEVGs in 1986 and successfully developed an early prototype. They constructed a tubular scaffold composed of bovine aortic ECs, SMCs, collagen, and a thin polyester mesh. Different types of cells were spatially organized within distinct layers of the scaffold in this model: ECs on the luminal surface, SMCs in the middle layer, and bovine adventitial fibroblasts in the outermost layer. Although this study laid a critical foundation for the advancement of vascular tissue engineering, the use of synthetic materials lacking bioactivity, such as polyester, limited the graft’s capacity for further growth and tissue remodeling [118]. In 2001, Shin’oka *et al*. [119] carried out the first clinical trial of a cell-seeded TEVG. They implanted a biodegradable scaffold seeded with autologous vascular cells into pediatric patients with congenital heart disease, marking the first clinical translation of TEVGs [119]. However, the wider clinical application of TEVGs remains limited by factors such as long manufacturing cycles, high costs, thrombosis, and immunogenicity [120, 121]. Current research efforts to address these challenges mainly focus on improving the cell sources, as well as functional modifications [122, 123].

### 4.1 Cell Selection for TEVGs

Human blood vessels consist of three layers. The intima is mainly composed of ECs and prevents thrombosis, the media contains SMCs and provides elasticity and contractility, and the adventitia contains fibroblasts and serves to anchor the vessel. During TEVG fabrication, the cell source is therefore a key factor influencing functional performance [124].

#### 4.1.1 Endothelial Cells

ECs are essential components of TEVGs and exhibit strong antithrombotic properties in native vessels [125, 126]. During the fabrication of TEVGs, ECs are typically seeded onto the surface of the scaffold prior to implantation in a process known as *in vitro* endothelialization [20]. Moreover, a comparative study demonstrated that EC-seeded TEVGs contribute not only to the formation of a mature endothelial layer, but also promote development of the smooth muscle layer [127].

However, the sourcing of autologous ECs is challenging due to patient-specific limitations and difficulties in cell isolation [128, 129]. To address this, researchers have explored the use of HUVECs as an alternative [130]. Umbilical cord tissue provides an abundant supply of immature, highly proliferative cells that can form complex vascular networks within the host. Nevertheless, challenges in purifying and stabilizing HUVECs have limited their widespread application in vascular grafting. In addition to HUVECs, researchers are also investigating the use of less differentiated stem cells, such as induced pluripotent stem cells (iPSCs) or endothelial progenitor cells (EPCs), which can be induced to differentiate into ECs *in vitro* and subsequently used for TEVG endothelialization [125, 131, 132].

In addition to *in vitro* endothelialization, the inner surface of the vascular graft can also be modified to capture host cells and promote *in situ* endothelialization [2]. By modifying material surfaces with capture molecules that mimic natural homing factors, circulating EPCs can be effectively recruited [133]. Various ligands have been applied to material surfaces to allow them to directly capture EPCs from the patient’s bloodstream, including monoclonal antibodies [134], functional peptides [135, 136], and certain bioactive factors [137].

#### 4.1.2 Smooth Muscle Cells

The tunica media of blood vessels is primarily composed of SMCs and ECM, forming the main structural component of most vessels and playing a key role in regulating vascular contraction to control blood flow [138, 139]. The incorporation of SMCs into TEVGs is a key factor in ensuring their successful application. Current strategies include seeding SMCs onto surface-modified scaffolds, and introducing stem cells followed by their differentiation into SMCs [140, 141, 142, 143]. Ardila *et al*. [144] developed an electrospun scaffold loaded with transforming growth factor β2 (TGFβ2), which enabled the sustained release of low concentrations of TGFβ2 and promoted the proliferation and migration of SMCs seeded onto the scaffold. In addition, the function of SMCs is also influenced by mechanical stress induced by blood flow, including cyclic stretch and hydrostatic pressure [145]. Studies have confirmed that the magnitude of cyclic stretch can increase the expression of SMC-specific differentiation markers and enhance the proliferation of SMCs [124, 146]. Hydrostatic pressure can also increase the proliferation of SMCs and promote the expression of certain proteins in the ECM [147, 148].

#### 4.1.3 Fibroblasts

Fibroblasts are spindle-shaped cells primarily found in connective tissues [124]. They are capable of synthesizing ECM components, particularly collagen and elastin, and serve as the predominant cell type in the tunica adventitia of blood vessels [149, 150]. Fibroblasts can differentiate into myofibroblasts in response to environmental stimuli, effectively enhancing the mechanical strength of TEVGs [151]. They can be obtained from the skin and exhibit strong proliferative potential [152]. Torres *et al*. [153] generated ABVs by culturing human fibroblasts to form cell-assembled ECM sheets, which were then rolled and matured. These grafts were used clinically for dialysis and remained functional for up to 20 months [153].

#### 4.1.4 Human Induced Pluripotent Stem Cells

hiPSCs have stem cell functions and are obtained by inducing somatic cells to express stem cell factors [154, 155]. They can be guided to differentiate into various cell types, including SMCs [156, 157]. Compared to embryonic stem cells, hiPSCs exhibit lower immunogenicity and are more readily accessible [124]. In previous applications of hiPSC-TEVGs, the low degree of SMC differentiation led to insufficient ECM synthesis, as well as reduced mechanical strength and radial dilation after implantation [158, 159, 160]. In a study published in 2020, Luo *et al*. [132] reported that a culture medium containing only TGF-β1 (excluding platelet-derived growth factor-BB (PDGF-BB)), combined with mechanical stretching after static culture, effectively improved the survival of hiPSC-derived SMCs. The resulting optimized hiPSC-TEVGs exhibited mechanical strength comparable to that of the saphenous vein [132]. Nevertheless, the hiPSC-TEVGs produced by this method still lack mature elastin fibers, and their mechanical strength has not yet reached the biomechanical level of human arteries. Moreover, the potential risk of tumor formation associated with hiPSCs, safety concerns related to the cell reprogramming process, and high manufacturing costs still limit the clinical application of hiPSCs. Therefore, further research is urgently needed to improve the safety and cost of hiPSCs [125, 161, 162]. At present, various factor-based regulatory strategies can significantly improve the efficiency of hiPSC differentiation into vascular lineage cells. Dash *et al*. [163] demonstrated that a multi-stage process, in which hiPSCs are first differentiated into MSCs and then directed toward VSMCs, can expand the intermediate cell population and thereby facilitate efficient downstream SMC differentiation. In addition, the use of PDGF-BB alone markedly upregulates the expression of the SMC marker gene calponin, and when PDGF-BB is combined with shear stress, it can further enhance the orientation and functionality of hiPSC-derived VSMCs [160, 164].

### 4.2 Functional Modification of TEVGs

#### 4.2.1 Enhancing Anti-Thrombotic Properties

Thrombosis is a major challenge for small-diameter vascular grafts [165]. The inflammatory response triggered by vascular grafts can lead to platelet aggregation, ultimately resulting in thrombus formation and graft occlusion [166]. Heparin, a potent anticoagulant used to prevent acute thrombosis after vascular implantation, has been widely applied as a coating on the inner layer of TEVGs through various methods [167]. Heparin has also been shown to promote the proliferation of ECs within TEVGs [168], and NO secreted by ECs plays an important role in inhibiting platelet aggregation [169]. The surface density of heparin immobilized on PU–PEG–Hep grafts was reported as 1.21 ± 0.29 µg/cm^2^, retaining only ~22% of the anticoagulant activity of free heparin, yet still achieving a 71.4% patency rate at 60 days in a rabbit model. Yang *et al*. [170] developed an AuNP–ChOX–Arg cascade system that catalyzed cholesterol into NO, reaching 18.95 µmol⋅L^-1^ within 20 min at a flux of 4.5 × 10^-10^ mol⋅cm^-2^⋅min^-1^, comparable to endothelial cells. In a hyperlipidemic rat carotid model, it markedly reduced platelet adhesion, an effect reversed by 2-(4-carboxyphenyl)-4,4,5,5-tetramethylimidazoline-1-oxyl-3-oxide (carboxy-PTIO), and maintained graft patency for 60 days [170].

#### 4.2.2 *In Situ* Endothelialization

**Fig. 2.**
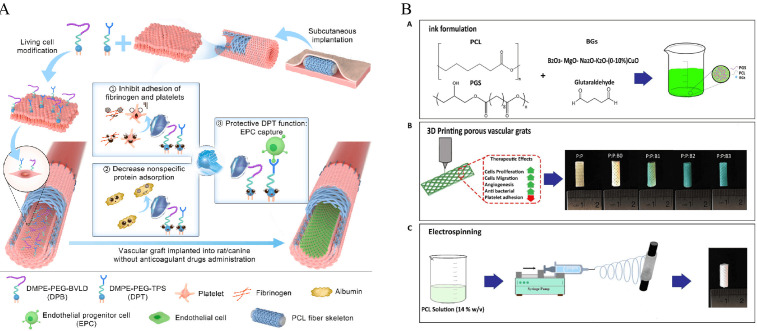
**Schematic representations of dual-modified PB vascular grafts and bilayer 3D-printed vascular scaffolds**. (A) Combined modification of the luminal surface of *in vivo* engineered PB grafts with DPB and DPT significantly promoted *in situ* endothelialization without the need for anticoagulant therapy [135]. (B) Schematic illustration of the construction of a bilayer vascular graft. The inner layer consists of a 3D-printed porous structure composed of PGS, PCL, and modified bioactive glass (BG) to accelerate vascular remodeling [181]. EPC, endothelial progenitor cell; PB, poly(butylene)-based; DPB, 2,2^′^-dithiodipyridine–based biofunctional linker; DPT, dual-peptide treatment; PGS, poly(glycerol sebacate).

Endothelial cells can secrete bioactive substances such as NO and heparin to maintain vascular patency [171]. Section 4.1.1 provided a preliminary introduction to the construction of an endothelial layer on TEVGs. Among the current strategies, *in situ* endothelialization has become a major focus of research as it avoids the need for *ex vivo* isolation and cultivation of ECs [172]. Yan *et al*. [135] used 1,2-dimyristoyl-sn-glycero-3-phosphoethanolamine-N-[poly(ethylene glycol)] (DMPE-PEG) as a lipid-anchoring structure to immobilize an EPC-capturing peptide on the luminal surface of TEVGs, significantly enhancing the adhesion of EPCs under dynamic flow. The DP structure also reduced nonspecific plasma protein adsorption, preventing the masking of EPC-binding sites (Fig. 2A) [135]. Platelet-rich plasma (PRP) is enriched with VEGF, TGF-β1 and PDGF, and effectively recruits EPCs to accelerate endothelialization [173, 174]. Li *et al*. [175] developed a PRP-poly(L-lactic acid) (PLLA)/gelatin composite scaffold that achieved an intimal coverage rate of 80.17% after one week in a rabbit common carotid artery transplantation model. Moreover, a 5% PRP-loaded scaffold achieved an intimal coverage of approximately 80% at 1 week and over 90% at 4 weeks, with functional eNOS⁺ endothelial cell coverage reaching 95%. *In vitro*, 2–5% PRP significantly promoted vascular endothelial cells (VECs) proliferation (EdU⁺: ~60% vs. 36%) and migration (32 vs. 10 cells per field), whereas excessive concentrations (10%) suppressed the growth of both VECs and SMCs, showing a dose-dependent effect. PRP-TEVGs were able to continuously release growth factors for over 25 days, with VEGF as the predominant component [175]. Current research primarily aims to enhance endothelialization by loading growth factors such as VEGF and fibroblast growth factor (FGF) [20]. However, these strategies are costly and have poor stability [176]. Some researchers have also explored the regulation of bioactive ion release as an alternative to growth factors [177]. Bioactive glass (BG) is an inorganic silicate material with excellent biocompatibility and bioactivity [178]. Different types of BG can be doped with various ions to exert distinct biological functions [179]. However, certain components commonly found in typical BGs (e.g., Ca, Si, and P) can induce coagulation, thus limiting the application of BGs in vascular grafts [180]. Alasvand *et al*. [181] developed Cu-doped BGs with Ca, P, and Si removed. This resulted in enhanced EC proliferation, migration, and VEGF secretion, while also providing strong antibacterial effects (Fig. 2B).

#### 4.2.3 Prevention of Endothelial Hyperplasia

Following TEVG implantation, SMCs may proliferate abnormally at the anastomosis, leading to intimal injury and vascular stenosis [182, 183]. Surface modification of TEVGs with drug-loaded coatings is a common strategy to address this issue [184]. Various agents including NO, small peptides, and traditional Chinese medicine compounds have been widely used to prevent SMC-related endothelial hyperplasia [185, 186]. Portulaca flavonoid (PTF) is a drug that can inhibit the PDGF-induced abnormal proliferation of SMCs. Xie *et al*. [187] employed PCL scaffolds loaded with PTF, which effectively suppressed SMC proliferation without cytotoxicity.

### 4.3 Animal Models and Clinical Translation of TEVGs

Mice and rats are the most commonly used experimental animals in current preclinical studies of TEVGs [188]. However, due to significant differences in physiological mechanisms, immune environments, and hemodynamics compared to humans, the experimental results obtained from these small animal models may still differ from actual clinical outcomes [20]. Large animals such as dogs, pigs, sheep, and baboons are widely used in TEVG research due to their anatomical structures and hemodynamic characteristics being similar to those of humans [189]. Computational fluid dynamics (CFD) studies have revealed significant interspecies differences in hemodynamic parameters. For example, in porcine coronary arteries, wall shear stress (WSS), time-averaged WSS (TAWSS), and relative residence time (RRT) are largely comparable to humans, but oscillatory shear index (OSI) differs markedly. In pulmonary artery models, sheep exhibit TAWSS values very close to humans (median ~1.5 Pa vs. 1.2 Pa; *p* = 0.42), whereas pigs show significantly higher TAWSS (~1.7 Pa; *p* < 0.05) [190]. However, both pigs and sheep demonstrate OSI values that differ significantly from humans. Therefore, specific interspecies differences must still be considered when using large-animal models. What’s more, sheep exhibit a stronger tendency toward coagulation [127], while pigs are more difficult to handle and also grow very rapidly, making them less suitable for long-term studies [191]. Non-human primates, such as baboons, closely resemble humans in terms of anatomy and thrombotic behavior [192, 193, 194], but their use is limited by the high costs and ethical concerns associated with animal experimentation.

To date, the application of TEVGs in humans remains limited. Shin’oka *et al*. [195] conducted a clinical study in which TEVGs composed of biodegradable scaffolds combined with autologous bone marrow cells were used to repair congenital heart defects in 25 pediatric patients. The median follow-up period was 11 years, during which no graft-related deaths were reported. However, 7 patients developed asymptomatic graft stenosis [195, 196]. Bockeria *et al*. [197] directly implanted cell-free biodegradable vascular grafts into five patients. Postoperative follow-up revealed favorable structural and functional outcomes for the grafts, with no graft-related adverse events reported [197]. Gutowski *et al*. [198] evaluated decellularized TEVGs seeded with human vascular SMCs for peripheral artery bypass in 20 peripheral artery disease (PAD) patients with SFA occlusion. Over 24 months, 26 graft-related complications occurred in 10 patients, but no amputations, infections, or aneurysmal changes were observed. The primary and secondary patency rates were 58% and 74%, respectively [198].

In recent years, great advances have been made with TEVGs and they have become a key focus of tissue engineering. However, to truly solve the clinical challenges presented by small-diameter grafts—like low long-term patency rates and limited treatment success—better materials and more reliable manufacturing methods must first be developed. Moreover, it is crucial that such grafts be thoroughly tested in large animal models and clinical trials to verify their safety and effectiveness before they can be widely used in patients.

### 4.4 Challenges and Barriers to the Clinical Translation of TEVGs

Although TEVGs have demonstrated potential in animal experiments and early clinical studies, they still face numerous unresolved challenges. The inability to ensure long-term patency remains the primary limitation hindering their clinical application. Early stenosis typically occurs within a few weeks after implantation, as most biomaterials used in TEVGs possess hydrophobic and negatively charged surfaces that can activate the intrinsic coagulation pathway, leading to acute thrombosis [199, 200, 201]. In addition, biofilm formation resulting from bacterial adhesion may also contribute to early stenosis of TEVGs [202]. Late stenosis is mainly caused by incomplete endothelialization and fibrosis with intimal thickening induced by persistent foreign body stimulation from the graft [203].

Insufficient mechanical performance and compliance are critical factors restricting the clinical application of TEVGs. In the human body, blood vessels are constantly subjected to multiple complex physiological forces, including blood flow pressure, pulsatile pressure, and external forces from surrounding tissues [204]. Therefore, artificial blood vessels must possess adequate mechanical strength and compliance to maintain stable elastic expansion and contraction under such conditions [25, 205]. However, current TEVGs often exhibit significant compliance mismatch compared with autologous vessels. This mechanical discrepancy at the anastomosis can lead to disturbed blood flow, aggravating platelet aggregation and fibrin thrombus formation [206]. Post *et al*. [207] demonstrated using an *in vitro* model that compliance mismatch not only reduces wall shear stress but also induces intimal thickening, thereby severely compromising long-term patency.

Besides, TEVGs usually require long periods of *in vitro* culture and preparation to ensure their mechanical strength, which also represents a major obstacle to their clinical translation [208]. Although TEVGs have shown promise in both preclinical and early clinical studies, long-term patency and mechanical compliance remain major barriers to clinical translation. The outcomes of completed clinical trials, summarized in Table 2 (Ref. [195, 196, 197, 198]), further illustrate these challenges in different patient populations and graft designs.

**Table 2.** **Completed clinical studies of TEVGs**.

	Patients (n)/Age	Indication	Implantation site	Graft type	Diameter	Follow-up	Patency/Interventions	Refs
Bone marrow cell–seeded TEVG	n = 42; 1–24 y	Complex CHD	Fontan conduit	PGA/PLLA	12–24 mm	Mean 16.3 months (max 31.6 months)	No occlusion	[195]
				PLCL				
Bone marrow cell–seeded TEVG	n = 25; 1–24 y	Single-ventricle CHD	Fontan conduit	PLA (n = 12)	12–24 mm	Mean 5.8 years (4.3–7.3)	6/25 stenosis (24%); 4 balloon dilatations, 1 stent; 1 mural thrombus	[196]
				PGA (n = 13)				
UPy-PCL TEVG	n = 5; 4–12 y	Single-ventricle CHD	Fontan conduit	UPy-PCL	18–20 mm	12 months	No stenosis; 2 APC occlusions (non-graft related)	[197]
Human acellular vessel	n = 20; 54–79 y	PAD SFA occlusion (TASC B/C)	Femoral–popliteal/SFA bypass	Decellularized human ECM (types I/III collagen, FN, VN)	6 mm	Mean 20.7 months (up to 24 months)	12-mo: 63% primary, 84% secondary; 24-mo: 58% primary, 74% secondary; 6/20 interventions	[198]

TEVG, tissue-engineered vascular grafts; ECM, extracellular matrix; CHD, congenital heart disease; PGA, poly(glycolic acid); PLLA, poly(L-lactic acid); PLCL, poly(L-lactide-co-ε-caprolactone); FN, fibronectin; VN, vitronectin; APC, anticoagulant protein C.

## 5. Emerging Technologies

In recent years, various emerging technologies such as engineered cells, artificial intelligence and 4D printing have been applied to the construction of vascular grafts. ECs exhibit remarkable immunological plasticity. In their quiescent state, ECs display low immunogenicity and secrete anti-inflammatory mediators such as NO, which also inhibit platelet aggregation [209]. The maintenance of a stable endothelial phenotype is therefore essential for ensuring the long-term patency and immune tolerance of TEVGs. Park *et al*. [210] reported that physiological shear stress induced hiPSC-ECs into a quiescent, anti-inflammatory phenotype, with increased antithrombotic factors (endothelial nitric oxide synthase (eNOS), krüppel-like factor 2,4 (KLF2, KLF4)) and reduced pro-thrombotic molecules (intercellular adhesion molecule-1 (ICAM-1), vascular cell adhesion molecule-1 (VCAM-1)). The implanted hiPSC-ECs were gradually replaced by host ECs, indicating their role as a temporary immunomodulatory scaffold that facilitates host cell repopulation [210].

Scaffold design and printing precision likewise represent critical challenges to the clinical translation of TEVGs. Advances in artificial intelligence provide promising avenues to address these limitations: large language models (LLMs) and graph neural networks (GNNs) have been employed to predict the mechanical properties, degradation behavior, and immunogenic potential of candidate polymers such as PLGA and PCL; reinforcement learning (RL) algorithms enable dynamic optimization of key bioprinting parameters, including extrusion speed and layer thickness, thereby more accurately replicating the physiological architecture of native vessels [211]. Moreover, the integration of computer vision with machine learning allows real-time monitoring and correction of defects during the printing process [212].

4D printing is based on 3D printing and integrates responsive (smart) materials, enabling grafts to respond to external stimuli in a controlled manner [213]. Baruch *et al*. [214] introduced the use of dynamic vascular wall bioinks (ECM–PNIPAM hybrid hydrogels), which undergo selective shrinkage at physiological temperature, enabling pre-designed vessels within cardiac parenchyma to spontaneously contract to capillary dimensions. This strategy realizes programmable *in situ* “scaling down” of microstructures within organs [214]. This printing strategy can be integrated with shear stress conditioning in bioreactors and endothelial functional coatings (such as NO donors or anticoagulant peptides), holding promise for further improving the long-term patency of TEVGs.

## 6. Conclusion and Prospects

Research on ABVs demonstrates the broad prospects in this field. In terms of material selection, the strengths and limitations of different material options must first be fully evaluated. Natural biomaterials have excellent biocompatibility, but present challenges in terms of mechanical durability and controlled degradation. In contrast, synthetic materials such as PET and ePTFE, while mechanically strong, often induce thrombosis, inflammation, and intimal hyperplasia, limiting their wider application. Innovative manufacturing techniques, especially electrospinning, 3D printing, TIPS, and microfluidic spinning, can enable precise microstructural control, mechanical regulation, and enhanced biointegration. These approaches facilitate better integration of cells and bioactive factors to more closely mimic the natural vascular architecture and significantly improve the performance of graft materials.

TEVGs have shown great potential in small-diameter vascular reconstruction. They have tunable mechanical properties, enhanced endothelialization capacity, and low reliance on *in vitro* cell seeding. However, challenges remain in terms of suitable cell sources. Although autologous cells are ideal, their limited availability and expansion potential make it necessary to explore stem cells and their derivatives. However, significant obstacles remain in terms of differentiation efficiency, immunogenicity, and reproducibility.

Surface modification and targeted drug delivery are essential to enhance TEVG functionality, especially in terms of antithrombotic, antimicrobial, and anti-inflammatory properties. Careful selection of animal models can greatly influence the relevance of preclinical studies. While small animals are advantageous for early studies, large animals provide deeper physiological insights for long-term evaluations.

Although early clinical trials of TEGVs have demonstrated short-term safety and efficacy, further improvement is still needed in terms of long-term patency and graft-related complications. Future studies should prioritize the use of standardized cell sources, optimized materials, integrated structure-function design, and rigorous clinical data, which are the key steps towards routine clinical application.
